# Monkey Malaria in a European Traveler Returning from Malaysia

**DOI:** 10.3201/eid1409.080170

**Published:** 2008-09

**Authors:** Anu Kantele, Hanspeter Marti, Ingrid Felger, Dania Müller, T. Sakari Jokiranta

**Affiliations:** Helsinki University Central Hospital, Helsinki, Finland (A. Kantele); University of Helsinki, Helsinki (A. Kantele, T.S. Jokiranta); Swiss Tropical Institute, Basel, Switzerland (H. Marti, I. Felger, D. Müller); Helsinki University Central Hospital HUSLAB, Helsinki (T.S. Jokiranta)

**Keywords:** *Plasmodium knowlesi*, malaria, monkey diseases, parasitology, PCR, emerging, communicable diseases, dispatch

## Abstract

In 2007, a Finnish traveler was infected in Peninsular Malaysia with *Plasmodium knowlesi*, a parasite that usually causes malaria in monkeys. *P. knowlesi* has established itself as the fifth *Plasmodium* species that can cause human malaria. The disease is potentially life-threatening in humans; clinicians and laboratory personnel should become more aware of this pathogen in travelers.

Traditionally, only 4 *Plasmodium* species have been known to cause malaria in humans: *P. falciparum*, *P. vivax*, *P. ovale,* and *P. malariae,* although >26 *Plasmodium* species are known to circulate among primate populations (*1*). Some of these species have been implicated in symptomatic human malaria after experimental or accidental infection (*2*). Only a few reports of naturally acquired monkey malaria in humans are currently available (*1*,*3*–*9*). The lack of data may be because light microscopy has been used as the sole diagnostic method and an atypical *Plasmodium* species may have been misidentified as one of the 4 traditional *Plasmodium* species causing human malaria.

*P. knowlesi* was first described in 1931 in a long-tailed macaque imported from Singapore to India; in 1932, *P. knowlesi* was experimentally shown to be infectious to humans (*10*). The first natural infection of *P. knowlesi* in humans was reported in 1965 in a man returning to the United States after a visit to Peninsular Malaysia (*11*). Subsequently, in 1971, there was a report of a presumed natural infection in a citizen of Malaysia (*6*). Despite extensive studies in Malaysia in the 1960s (*2*), no other reports were published on naturally acquired *P. knowlesi* infections in humans until 2004, when Singh et al. studied PCR-negative *P. malariae* cases in the Kapit division in Sarawak, Malaysia (*3*). A different PCR analysis showed that *P. knowlesi* caused 58% of the 208 malaria cases studied. Further cases reported from China (*4**)*, Thailand (*5*), Philippines (*8*), and Singapore (*12*) show that *P. knowlesi* infections in humans are not found exclusively in Malaysia. Recently, Cox-Singh et al. reported that *P. knowlesi* is widely distributed among inhabitants of Malaysia (*7*).

## The Study

A 53-year-old Finnish man was admitted to a local hospital in Finland in March 2007 with fever after 4 weeks of travel in Peninsular Malaysia. He had not taken any antimalarial prophylaxis. In Malaysia, he spent 2 weeks in Kuala Lumpur and made a few day trips to surrounding rural areas. Thereafter, he traveled by car to the northwestern coast and stayed for 5 days in the jungle ≈80 km south of Ipoh. While in this area, he slept in a house without mosquito screens or nets and did not use any repellents; he did not report any mosquito bites. The last week of his travel was spent in the Langkawi Beach area where he stayed at a high-quality hotel. During his trip he occasionally had some minor abdominal problems, but these symptoms subsided spontaneously after his return to Finland. High fever (38.8^o^C axillary temperature) occurred 3 days after his return to Finland but abated quickly. On the fourth day, the fever returned and he sought medical care at a local hospital. Laboratory tests showed the following results: C-reactive protein 2.0 mg/dL (normal range <1.0 mg/dL), hemoglobin 15.2 g/dL (normal range 13.4–16.7 g/dL), leukocyte count 2.6 *×* 10^9^/L (normal range 3.4–8.2 *×* 10^9^/L), and thrombocytes 143 *×* 10^9^/L (normal range 150–360 *×* 10^9^/L). Blood smear was positive for *Plasmodium* organisms, and the causative agent was identified as *P. falciparum* with levels of parasitemia <1.0%. The patient was admitted to the hospital and given intravenous (IV) quinine dihydrochloride and oral doxycycline.

On day 2 of the patient’s hospital stay, fever returned and he was transferred to the Helsinki University Central Hospital (Department of Infectious Diseases at Aurora Hospital). Blood smears obtained there showed *Plasmodium* parasites that were considered atypical, and the laboratory reported suspicion of a co-infection (*P. falciparum* and *P. malariae*) (Figure). The IV quinine dihydrochloride was replaced with oral quinine hydrochloride, and doxycycline was continued. During treatment, the patient experienced an attack of hypoglycemia (electrocardiogram and blood pressure was normal during this attack), transient mild visual and hearing loss, and transient lymphopenia (a low of 0.46 *×* 10^9^/L). He received quinine hydrochloride and doxycycline for a total of 10 days.

**Figure:**
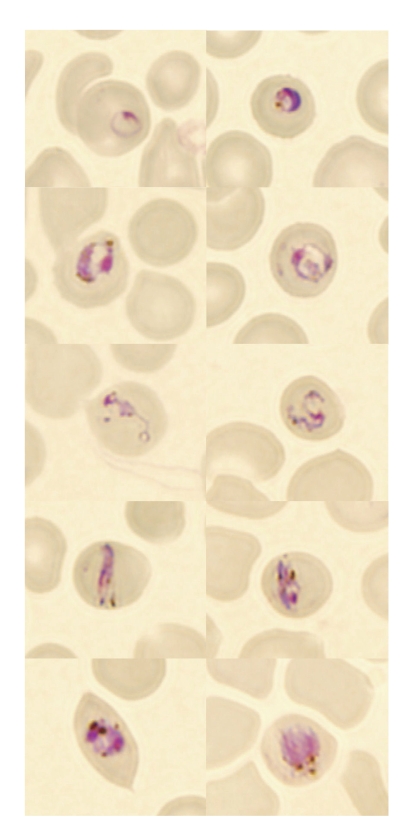
Microscopic findings in the thin blood smears of a patient with *Plasmodium knowlesi* malaria. Early ring forms are shown in the first row, later trophozoites in the second and third rows, trophozoites resembling band forms in the fourth row, and putative early gametocytes or schizonts in the fifth row. Size of the infected erythrocytes is normal. Antimalarial medications, given 8 hours before the blood shown in the smear was drawn, could have affected morphology. (Original magnification ×1,000.)

Because identification of the *Plasmodium* species was difficult, a blood sample was drawn for PCR analysis on day 2 of hospitalization. First, a nested PCR was performed according to a standard protocol with rOva1 and rPLU2 primers (template DNA purified in Basel from 200 µL of erythrocytes by QIAamp DNA Mini Blood Kit (QIAGEN, Helsinki, Finland) (*13*,*14*), but the reaction did not yield any amplification product. Nested PCR was repeated with an alternative primer pair (rPLU6 and rPLU2) (*14*) derived from a conserved region of the 18S rRNA marker gene, and an amplicon was obtained. Failure of PCR amplification has been reported for some *P. ovale* isolates (*15*); therefore, a *P. ovale* infection was suspected, and the patient was given primaquine phosphate for 14 days as an outpatient to eradicate possible liver hypnozoites. The PCR product was subjected to direct nucleotide sequencing (GenBank accession no. FJ009511) and found to be identical to 2 *P. knowlesi* sequences previously submitted to GenBank, 1 human isolate from Malaysian Borneo (AY327556) and a *Macaca mulatta* isolate from Columbia (U72542). Six other published *P. knowlesi* sequences differ from our sequence only by 1 nucleotide (99% identity). In contrast, a number of differences were seen between our sequence and the *P. ovale* sequences (*15*). The sequence from our case showed only 50% identity to the ova1e primer; therefore, we concluded that our patient was infected with *P. knowlesi*. During the 12-month follow-up period, the patient showed no signs of relapse.

## Conclusions

We suggest that *P. knowlesi* infection should be considered in malaria patients who have a history of a travel to forested areas in Southeast Asia, especially if *P. malariae* malaria is diagnosed or atypical plasmodia are seen with microscopy. The asexual stages of various species of *P. knowlesi* can easily be misidentified as *P. malariae* in light microscopic examination (Figure) (*3*,*7*,*10*). Because most laboratories diagnose malaria by light microscope examination only, numerous cases of *P. knowlesi* malaria may have been misdiagnosed as ordinary *P. malariae* malaria; monkey malaria may be more widespread among humans than was previously thought. As the disease is potentially dangerous, a proper identification of the malaria species is crucial. If PCR assays for malaria detection are used, PCR primers specific for *P. knowlesi* (*3*) should be included to provide valuable diagnostic information.

*P. knowlesi* has established itself as the fifth species of *Plasmodium* that causes human malaria (*3*,*7*,*12*). Because the disease is potentially life-threatening in humans, laboratory clinicians and physicians (especially those taking care of travelers) should become more aware of this disease; it is easily misdiagnosed as a less severe form of malaria.
